# Collagen–Chitosan Composites Enhanced with Hydroxytyrosol for Prospective Wound Healing Uses

**DOI:** 10.3390/pharmaceutics17050618

**Published:** 2025-05-06

**Authors:** Miguel P. Batista, Margarida Pimenta, Naiara Fernández, Ana Rita C. Duarte, Maria do Rosário Bronze, Joana Marto, Frédéric Bustos Gaspar

**Affiliations:** 1 iBET—Instituto de Biologia Experimental e Tecnológica, Apartado 12, 2781-901 Oeiras, Portugal; mbatista@ibet.pt (M.P.B.); margarida.pimenta@ibet.pt (M.P.); naiara.fernandez@ibet.pt (N.F.); mbronze@ibet.pt (M.d.R.B.); 2 LAQV-REQUIMTE, Associated Laboratory for Green Chemistry—Network of Chemical and Technology, Departamento de Química, Faculdade de Ciências e Tecnologia, Universidade Nova de Lisboa, Quinta da Torre, 2829-516 Caparica, Portugal; ard08968@fct.unl.pt; 3 Instituto de Tecnologia Química e Biológica António Xavier, Universidade Nova de Lisboa, Av. da República, 2780-157 Oeiras, Portugal; 4 FFULisboa, Faculty of Pharmacy, Universidade de Lisboa, Av. das Forças Armadas, 1649-019 Lisboa, Portugal; 5 Research Institute for Medicines (iMed.ULisboa), Faculty of Pharmacy, Universidade de Lisboa, Av. das Forças Armadas, 1649-003 Lisboa, Portugal

**Keywords:** collagen, chitosan, hydroxytyrosol, biomaterials characterization, advanced wound dressings, biomedical applications

## Abstract

**Background/Objectives:** Recent studies highlight the excellent wound-healing properties of collagen and chitosan materials. Combining these polymers with a bioactive compound could enhance their effectiveness as next-generation wound dressings. Hydroxytyrosol (HT), an antioxidant derived from olive oil, may aid wound healing due to its anti-inflammatory, antimicrobial, and angiogenesis-stimulating properties, making it a beneficial addition to collagen–chitosan dressings. It could be a beneficial addition to collagen–chitosan dressings, thus improving their therapeutic effects. This study screens the potential of collagen–chitosan composites with HT for wound-healing applications and assesses the influence of the compound’s incorporation on the materials’ properties. **Methods:** The material production involved incorporating chitosan and HT into a marine collagen extract. The resulting collagen–chitosan–HT material was obtained through freeze-drying. Prototype dressing characterization included morphology by scanning electron microscopy, solid and hydrated state by textural and rheological studies, and in vitro HT release studies. The materials’ cytocompatibility screening was assessed using a mouse fibroblast cell line, and the antibacterial activity was evaluated against microorganisms commonly implicated in wound infections. **Results:** Burst strength, viscosity, frequency sweep test, tackiness, and adhesion results indicate that chitosan contributes to the material’s mechanical robustness by maintaining a high viscosity and preserving the material’s gel structure. The in vitro release studies suggest an HT-controlled release profile with a maximum release (70%) achieved after 10 h. Biological experiments confirmed the materials’ cytocompatibility with skin cells and very promising antibacterial efficacy against *Staphylococcus aureus* and *Pseudomonas aeruginosa*. **Conclusions:** In conclusion, HT was successfully incorporated into a collagen–chitosan matrix, enhancing the therapeutic prospect of the resultant material. The collagen–chitosan–HT composite presents a promising potential as an advanced wound-healing material.

## 1. Introduction

Wound management remains a critical challenge in clinical practice, with various types of wounds requiring tailored approaches for effective healing. Acute wounds, such as surgical incisions and traumatic injuries, typically follow a predictable healing course. In contrast, chronic wounds, including diabetic ulcers, pressure sores, and venous leg ulcers, often exhibit prolonged inflammation and impaired healing, posing significant burdens on patients and healthcare systems [1,2]. The wound-healing process is a complex and dynamic sequence of events that involves four overlapping phases: hemostasis, inflammation, proliferation, and remodeling [3]. Traditional wound dressings, such as gauze and bandages, primarily serve as protective barriers. However, they often fail to address the complex biological processes of wound healing. Understanding wound healing has led to the development of a new generation of bioactive dressings. These dressings are not just physical barriers but active participants in the healing process. They deliver therapeutic agents, maintain a moist wound environment, and promote cellular activity essential for tissue repair [1,4].

Furthermore, these dressings may present several forms, including hydrated-state hydrogels, films, and hydrocolloid dressings or solid-state fibers, acrylics, and foams, each tailored to specific wound types and healing stages [4]. Despite some limitations, such as difficulty in application and removal due to limited flexibility, solid wound dressings offer several advantages over hydrogels and other hydrated materials in wound management. They are typically easier to sterilize and handle and do not require special storage conditions. Due to the low moisture content, they can absorb exudate efficiently, maintaining an optimal wound moisture balance in highly exudative wounds [5]. Solid-state materials also provide a longer shelf life, are less prone to microbial contamination, and prevent the degradation of unstable bioactive compounds.

Emergent new-generation solid dressings comprise various synthetic and natural polymeric matrices. Synthetic polymers include poly(lactic-co-glycolic acid), polyurethane, and polycaprolactone. On the other hand, natural polymers such as alginate, chitosan, and collagen are also frequently explored as wound dressing components [4,5]. Some natural polymers possess inherent bioactive properties and offer several advantages over synthetic compounds as they are biocompatible and biodegradable, reducing the risk of adverse reactions. Moreover, several natural polymers, such as chitosan and collagen, can be derived from undervalued food industry by-products, potentially making the wound dressing production process more sustainable and eco-friendlier [6,7]. Collagen, a primary structural protein in the human extracellular matrix, provides a scaffold that supports cell adhesion, migration, and proliferation, thereby facilitating tissue regeneration [8]. Several works have mainly presented the potential of marine collagen due to its excellent wound-healing properties and abundant sources [7,9,10]. On the other hand, chitosan has also emerged as a promising polymer for wound dressings, showing biocompatibility, biodegradability, and antimicrobial activity properties [6]. Additionally, chitosan could significantly enhance the mechanical robustness of wound dressing materials, ensuring resistance to physical stresses without tearing or losing integrity.

Incorporating bioactive compounds in novel polymeric dressings could enhance the wound-healing process and therapeutic outcomes. These bioactive agents, such as antimicrobial peptides, growth factors, and anti-inflammatory molecules, actively participate in the healing process by promoting cell proliferation, reducing infection risks, and modulating the inflammatory response [4,5,11]. Hydroxytyrosol (HT), a phenolic compound typically found in olive oil products and processing sub-products, has garnered significant attention for its potential in wound healing applications. Its distinctive antioxidative properties help mitigate oxidative stress at the wound site, thereby protecting cells from damage and promoting tissue repair [12]. HT also exhibits anti-inflammatory and antimicrobial activities, which are relevant for reducing inflammation and preventing infections during wound healing [12,13]. Several studies have also shown that HT can enhance angiogenesis and collagen synthesis, further accelerating wound closure and improving overall healing outcomes [13,14]. These diverse advantages position HT as a promising bioactive compound that can be incorporated into advanced wound care products.

This work explores collagen–chitosan composites’ production and wound-healing potential enhanced with HT for topical applications. The authors intend to combine marine collagen’s structural and regenerative properties with chitosan’s antimicrobial and mechanical robustness, together with the incorporation of HT, to further enhance the therapeutic efficacy of the composites. This study evaluates the morphology, the solid- and hydrated-state mechanical properties, and the HT release profile of collagen–chitosan composites. Bioassays were also conducted to determine the toxicity screening of the skin cells’ contact and assess the compound’s synergic antimicrobial activity against microorganisms recognized as skin commensals and nosocomial pathogens.

## 2. Materials and Methods

### 2.1. Materials

HT (≥98%) was supplied by Greenway Biotech Co., Ltd. (Suzhou, China). Low-molecular-weight (50–190 kDa, 75–85% deacetylated) chitosan, Eagle’s minimum essential medium (EMEM), phosphate-buffered saline (PBS), pH = 7.4, dimethyl sulfoxide (DMSO), and hydrochloric acid (HCl, ≥37%) were purchased from Sigma (St. Louis, MO, USA). Nonessential amino acids (NEAA), fetal bovine serum (FBS), and 0.25% (*w*/*v*) Trypsin-EDTA were purchased from Gibco (Life Technologies, Waltham, MA, USA). CellTiter 96^®^ Aqueous Non-Radioactive Cell Proliferation Assay (MTS) reagent assay was obtained from Promega (Madison, WI, USA). Tryptic soy broth (TSB) was purchased from Scharlau (Barcelona, Spain), and tryptone soya agar (TSA) and nutrient agar (NA) were purchased from Oxoid (Hampshire, UK). Cation-adjusted Mueller Hinton broth (CAMHB) was acquired from BD Difco (Franklin Lakes, NJ, USA). Antibiotic test discs and SnakeSkin™ dialysis tubing (10 kDa) were purchased from Fisher Scientific (Brussels, Belgium). All solvents used for the HT quantification studies were of HPLC grade. *Staphylococcus aureus* ATCC 6538 (WDCM 00193) and *Pseudomonas aeruginosa* ATCC 27853 (WDCM 00025) were the strains selected to represent Gram-positive and Gram-negative bacteria, respectively. These species are recognized as skin commensals as well as nosocomial pathogens [15,16].

### 2.2. Biomaterials Preparation

**Figure 1:**
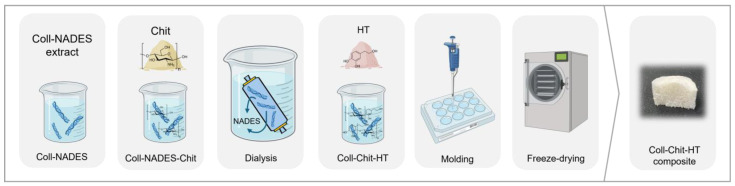
Schematic representation of the collagen (Coll)–chitosan (Chit)–hydroxytyrosol (HT) biomaterials production process.

The collagen extract used to produce the solid biomaterials in this study was obtained through extraction, as detailed in our previous work [7]. The extract consists of collagen (0.4 wt.%) and a natural deep eutectic solvent (NADES) composed of citric acid, xylitol, and water (1.0:1.0:10 mol ratio). Chitosan was added to the collagen extract in a 10:1.0 *w*:*w* (collagen:chitosan) ratio, leveraging the acidic nature of the solution required for chitosan solubilization. Chitosan was dissolved under magnetic stirring at room temperature for 2 h. Subsequently, the collagen-NADES extract and chitosan solution were dialyzed against distilled water to remove the NADES. Dialysis was performed for 5 days at 4 °C, with the solutions changed every 12 h [7]. After dialysis, HT was dissolved in the dialyzed collagen–chitosan solution for 15 min at a concentration of 1.6 mg/mL. Finally, the collagen–chitosan–HT solution was transferred into molds (12-well and 24-well plates) and gelled at 4 °C for 18 h. The hydrogels were frozen (−20 °C) and freeze-dried for 48 h. All freeze-dried samples were prepared in triplicate. The collagen (Coll)–chitosan (Chit)–hydroxytyrosol (HT) biomaterial production process is summarized in Figure 1.

### 2.3. Morphology

The morphology of the samples was analyzed using field emission gun scanning electron microscopy (SEM), using a Phenom ProX G6 Desktop SEM (Thermo Fisher Scientific, Waltham, MA, USA) at an accelerating voltage of 10 kV and working distances of 2–6 mm. The samples were imaged at several magnifications (50× and 100×), with the 100× magnification selected as the most suitable for observing the materials’ microstructure.

### 2.4. Mechanical Properties

#### 2.4.1. Textural Analysis

Before conducting textural analysis, the samples were pressed into discs to normalize the thickness of each material. Thickness normalization was carried out using a compressed air press, where each material was pressed three times for 5 s at 5 bars. The textural analysis measured the burst strength (BS) and distance to burst (DB) calculated from the puncture test. This test assesses the material’s strength against puncture by a cylindrical probe at a constant speed and the distance at the breaking point. This analysis was performed using a texture analyzer (TA.XT plus, Stable Microsystems, Waverley, UK). The samples were secured to a film-supporting rig (HDP/FSR) and compressed with a probe adaptor (AD/100) at a speed of 1 mm/s until rupture. A load cell of 30 kg was used, and the puncture test was carried out in triplicate.

#### 2.4.2. Rheology Studies

The rheological studies were conducted using a controlled stress Kinexus Lab + Rheometer (NETZSCH Analyzing and Testing, Selb, Germany) with the software rSpace for Kinexus (Version 1.76.2398.0, 2019, Selb, Germany) at 32 °C to mimic skin surface temperature. The solid samples were hydrated with PBS (pH = 7.4) in a 1:1 and 1:4 *w*:*w* ratio at room temperature for 15 min. This hydration was aimed at mimicking wound fluid exudate absorption. The hydrated samples were characterized in terms of viscosity, oscillation, tackiness, and adhesion. All methodologies were assessed using a smooth plate–plate geometry with an upper diameter of 20 mm (PU20). The shear rate range for viscosity measurements varied from 0.1 to 10 s^−1^. Regarding the oscillatory method, an amplitude sweep test was first performed to define the linear viscoelasticity range (shear strain was 1% for all samples). The oscillation frequency of the samples was determined using frequencies ranging from 0.1 to 10 Hz. For tackiness and adhesion measurements, a pull-away test was conducted with 0.5 mm/s for gaping speed, 20 mm for the final gap, and 0.5 mm for the working gap. The testing was performed in triplicate for viscosity and oscillation, while tackiness and adhesion were carried out with 5 replicates.

#### 2.4.3. In Vitro HT Release Studies

The release of HT from the Coll:Chit:HT biomaterial prepared was studied using Franz diffusion cells, each with a 4 mL receptor volume and a permeation area of 1 cm^2^. The cells were equipped with hydrophilic polysulfone membrane filters (Supor^®^ 450 PES Membrane Disc Filters, 0.45 µm, Pall Corporation, New York, NY, USA), with PBS as the receptor phase. Before conducting the experiment, the membranes were washed and equilibrated with the receptor phase, then placed between the donor and receiver compartments of the Franz diffusion cells. The system was maintained at 32 °C ± 1 °C for 30 min prior to the start of the experiment. The donor phase consisted of 62 mg of each biomaterial obtained after freeze-drying a solution containing a total HT-loaded content of 7.8 mg, quantified by HPLC-DAD. Each biomaterial replicate was evenly applied to the surface of the membrane in the donor compartment and was immediately sealed with Parafilm^®^ to prevent water evaporation. Samples of 200 µL were collected from the receptor phase at different intervals (1 h, 2 h, 4 h, 6 h, 10 h, and 24 h), with the collected volume immediately replaced by fresh receptor phase maintained at the same temperature. The studies were performed using four Franz cells, and the amount of permeated HT was determined using the HPLC method described below. The percentage of HT released into the medium was calculated using the following equation:

(1) $$\mathrm{Cumulative}\;\mathrm{release}\;\mathrm{percentage}\;=\sum_{t=0}^{t}\frac{M_{t}}{M_{0}}\times 100\;,$$

where M_t_ is the cumulative amount of HT released at each sampling time point t and M_0_ is the initial weight of the HT in the materials.

The data obtained from in vitro release studies were computed using DDsolver [17], which is an Excel plugin module, and the resultant data were fitted to different kinetic models:

(2) $$\mathrm{Zero}-\mathrm{order}\;\mathrm{kinetics}\;\;\;\;\;\;F=K_{0}\times t,$$

where K_0_ is the zero-order release constant;

(3) $$\mathrm{First}-\mathrm{order}\;\mathrm{kinetics}\;\;\;\;\;\;\;F=100\times (1-e^{{-K}_{1}\times t}),$$

where K_1_ is the first-order release constant;

(4) $$\mathrm{Higuchi}\;\mathrm{model}\;\;\;\;\;\;\;F=K_{H}\times t^{1/2},$$

where K_H_ is the Higuchi release constant;

(5) $$\mathrm{Korsmeyer}\text{–}\mathrm{Peppas}\;\mathrm{model}\;\;\;\;\;\;\;F=K_{\mathrm{KP}}\times t^{n}$$

where K_KP_ represents the release constant, which takes into account the structural and geometric characteristics of the drug-dosage form, and n is the diffusional exponent that indicates the drug-release mechanism.

In all models, F denotes the fraction (%) of the released drug over time, t. The adjusted coefficient of determination (R^2^ _adjusted_) was estimated for each model, fitted, and used to assess its ability to describe the dataset adequately. The R^2^ _adjusted_ values and the Akaike minimum information theoretical criterion (AIC) were used to evaluate the fit of the different models. The AIC method involves fitting the model to the existing data and analyzing its capacity to predict future values. When comparing several competing models, the model with the lowest AIC value is considered the best fit [18,19].

#### 2.4.4. HT Quantification by HPLC

Quantification of HT was conducted using an HPLC-DAD Vanquish (Thermo Fisher Scientific, USA) equipped with a quaternary pump, a solvent degasser, an autosampler (10 μL injection volume at 12 °C), and a column oven at 35 °C. The equipment was also coupled to a Photodiode Array Detector Waters 996 PDA (Waters, Wilmslow, UK), scanning wavelength absorption from 190 nm to 680 nm. The column used was a Luna 5 µm C18(2) 100 Å (250 × 4 mm). A gradient method was applied with eluent A (Milli-Q water with 0.5% formic acid) and eluent B (90% acetonitrile, 9.5% Milli-Q water, and 0.5% formic acid). A flow rate of 0.6 mL/min was set, and the following elution program was applied: 0–15 min 94.4% of A and 5.6% of B; 15–22 min 80% of A and 20% of B; 22–45 min 60% of A and 40% B; 45–55 min 100% of B; and finally returning to the initial conditions for 10 min [20].

### 2.5. Bioassays

Materials’ sterilization was performed for the biological assays and validated using ultraviolet (UV) irradiation and sterility confirmation tests following the methodology referenced in the literature [21].

#### 2.5.1. Cytocompatibility

To simulate the interaction of the materials with skin cells, cytocompatibility was evaluated using the direct contact method as outlined in ISO 10993-5:2009 [22], a sensitive approach for evaluating the toxicity of medical devices [21,22]. Mouse fibroblasts NCTC clone 929 (ECACC 88102702) cells, acquired from the European Collection of Authenticated Cell Cultures (ECACC, Public Health England, Salisbury, UK), were routinely cultured in the standard EMEM supplemented with 1% (*v*/*v*) NEAA and 10% (*v*/*v*) heat-inactivated FBS. Stock cells were maintained as monolayers in 75 cm^2^ culture flasks, subcultured weekly by seeding 30,000 cells/cm^2^, and incubated at 37 °C in a 5% CO_2_ humidified atmosphere. For cell passage, cells were detached upon reaching approximately 80% confluence using 0.25% (*v*/*v*) trypsin/EDTA at 37 °C. After collection, cell viability was assessed using the standard trypan blue-staining procedure, and cell numbers were counted with an automated cell counter (Invitrogen Countess™ 3, Thermo Fisher Scientific). All cellular assays were performed with cells between passages 10 and 20.

Cell viability was quantified using the MTS cytotoxicity assay, following the methodology described in the literature with slight modifications [21,22]. NCTC clone 929 cells were seeded into 24-well plates (0.6 mL volume) at a 3.0 × 10^4^ cells/cm^2^ density and incubated for 24 h (~1 doubling period) to develop a semi-confluent monolayer. After 24 h, the medium was replaced with fresh EMEM supplemented with 0.5% FBS, and following the direct contact method, the cells were then incubated for 24 h with the test samples. The materials for this assay were previously prepared in 24-well plates, at a sample amount of 32 mg/well. In line with ISO 10993-5, these sterilized samples were directly added into the seeded wells, achieving a final testing concentration of 53.3 mg/mL. Afterward, the cytocompatibility testing samples were removed, and the cells were rinsed three times with PBS before being incubated for 3 h with 0.6 mL of MTS reagent assay, and diluted according to the manufacturer’s instructions. Absorbance was measured at 490 nm using a microplate spectrophotometer (EPOCH, 219 Bio-Tek, Winooski, VT, USA). The experiments were conducted in triplicate over three independent assays. A cytotoxicity positive control was performed with a treatment of 10% (*v*/*v*) DMSO solution diluted in EMEM. The results were presented as a percentage of cellular viability (Viab.%) relative to the negative control (untreated cells, where no sample or cytotoxic compound was added). A cytotoxic effect was considered for viability percentages below 70%, according to ISO 10993-5.

#### 2.5.2. Antimicrobial Susceptibility Testing (AST)

Antimicrobial susceptibility testing (AST) assays followed the broth microdilution method outlined in the CLSI M07-A10 guidelines [23]. Compound stock solutions were added to a 96-well round-bottom microtiter plate and serially diluted twofold in CAMHB to achieve a range of concentrations. A standardized inoculum was prepared in CAMHB to ensure that approximately 5 × 10^4^ CFU were present in each well after inoculation. The inoculated microtiter plates were incubated aerobically at 37 °C for 24 h. Minimal inhibitory concentration (MIC) values were read as the lowest concentration of the compound that inhibited visible growth after 24 h of incubation. For each tested compound stock solution assayed, a growth control (CAMHB and diluted inoculum), a medium sterility control (CAMHB), and a stock solution sterility control (CAMHB and compound stock solution) were also included. All compound stock solutions were previously filter-sterilized (0.45 µm) using a solvent-compatible filter, and the results are expressed as median values of three biological replicates performed. The MIC values for the dissolving agent (HCl) were assessed to confirm that it did not inhibit bacterial growth under the assay conditions. A summary of all relevant compounds, dissolving agents, and the corresponding highest-tested concentrations is provided in Table 1.

#### 2.5.3. Antimicrobial Activity Determination of Biomaterials

The antibacterial performance of the biomaterials was assessed using the absorption method in conjunction with the quantification by plate count method from ISO 20743:2021, with some modifications [24]. All samples (biomaterials and the control fabric cotton disks) were prepared by weighing 20 mg of each and sterilizing them, as mentioned before. All sterile vials containing the samples were inoculated with 10 μL of a normalized bacterial suspension (1–3 × 10^5^ CFU/mL), allowing for complete absorption. Following inoculation and incubation steps (at 0 h and 24 h), the bacterial recovery involved adding 1 mL of shake-out physiological saline solution. This antimicrobial activity assay was conducted for the selected target bacteria, and the results are reported as the mean values from three biological replicates. The antibacterial activity value was calculated according to ISO 20743:2013, classifying efficacy as strong (for antibacterial activity value ≥ 3), significant (for 2 ≤ antibacterial activity value < 3), or negligible (for < 2 antibacterial activity value).

### 2.6. Statistical Analysis

The data was statistically analyzed with GraphPad Prism 10 (GraphPad Software, Inc., San Diego, CA, USA). All values were tested for normal distribution and equal variance. Once homogeneous variances were established, the data were analyzed using a one-way analysis of variance (one-way ANOVA) coupled with Tukey’s post hoc analysis to identify means with significant differences. Statistical differences were reported when at least the *p* < 0.05 condition was verified.

## 3. Results and Discussion

Previously published works have described the production of collagen materials with chitosan and bioactive compounds for biomedical applications [10,25,26,27,28,29,30,31,32,33,34,35]. This article builds upon these works and presents a new solid collagen material that leverages the NADES solubility properties of collagen, obtained according to our previous work [7], together with chitosan, while additionally incorporating a potent antioxidant (HT), which has been minimally studied as a biomaterial ingredient for potential biomedical applications. Three different materials composed of collagen (Coll), collagen and chitosan (Coll:Chit), and collagen with chitosan and HT (Coll:Chit:HT) were prepared to assess the influence of incorporating chitosan and HT in the collagen material. Chitosan was incorporated into the collagen extract in a 10:1.0 Coll:Chit *w*:*w* ratio, according to several solubilization tests indicating this maximum achievable chitosan incorporation. HT was incorporated based on the MIC obtained in the antimicrobial susceptibility testing performed against *S. aureus* and *P. aeruginosa* (Table 2). This test was also completed for chitosan to verify that both compounds used and incorporated into the collagen matrix were active antimicrobials against well-recognized skin commensals and nosocomial pathogens [15,16].

As expected, chitosan and HT displayed their antibacterial activity against both bacteria. This result agrees with the literature since, unlike collagen, chitosan and HT have a well-established bioactivity against these microorganisms [6,12,13,36,37,38]. HT is known to be unstable in aqueous media. It can degrade over time, especially when exposed to light and oxygen [39,40]. Nevertheless, preliminary testing revealed that aged HT solutions (24 h at 37 °C and 60 days at 4 °C) retained their antimicrobial activities since the MIC remained, at least, the same as that of a freshly prepared HT solution (Table S2).

**Figure 2:**
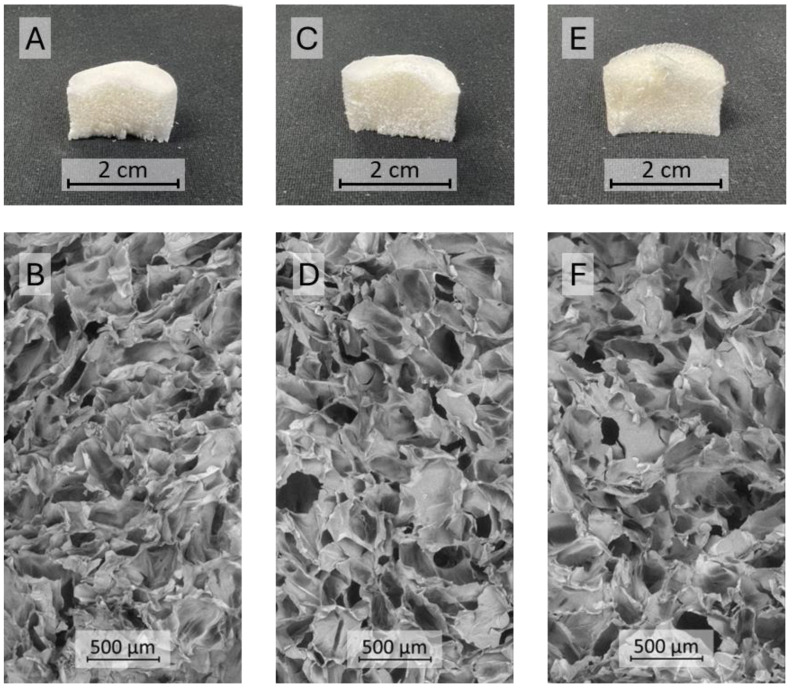
Cross-sectional photographs (**A**,**C**,**E**) and SEM micrographs (**B**,**D**,**F**) at 100× magnification of the different materials produced with collagen (Coll), chitosan (Chit), and hydroxytyrosol (HT): Coll (**A**,**B**), Coll:Chit (**C**,**D**), and Coll:Chit:HT (**E**,**F**).

Based on chitosan solubility tests and the highest HT MIC value recorded for both bacteria (1.6 mg/mL), a material composed of collagen, chitosan, and HT was obtained in a 10:1.0:1.2 Coll:Chit:HT *w*:*w*:*w* ratio. The Coll and Coll:Chit materials were obtained by performing the Coll:Chit:HT production process described in the materials and methods section without the HT (Coll:Chit) and the chitosan and HT incorporation steps (Coll). The resulting materials’ macroscale photographs and corresponding scanning electron microscopy (SEM) micrographs are shown to evaluate the morphology (Figure 2).

The resulting freeze-dried gels exhibited a similar monolithic macrostructure with a sponge-like appearance. SEM micrographs revealed that all the materials presented a comparable microstructure consisting of large macropores (pore diameter in the range of several μm) with dense areas between pores. Several studies reported that this structure is typical of freeze-dried collagen gel materials [9,41]. The similar structure among all produced samples suggests that chitosan and HT did not influence the different collagen materials’ morphology.

Materials for topical application should exhibit acceptable mechanical characteristics and must be easy to apply [42]. Therefore, mechanical robustness and malleability are important attributes of topical solid dressings for biomedical applications. These features ensure that dressings provide consistent protection against wounds’ external contaminants and maintain their structural integrity during handling [43,44]. In this context, several techniques were applied to determine relevant features that characterize the samples. First, a puncture test was performed to determine the material’s strength to be punctured by a cylindrical probe at a constant speed and the distance at the breaking point. This distance is defined as the length from when the probe contacts the material until it breaks [45]. The results of the textural analysis of the solid materials are presented in Table 3.

The analyzed materials showed burst strength values in the range of 13.9 N to 19.7 N. The solid sample composed solely of collagen presented a lower burst strength value, whereas samples with chitosan ruptured at higher force values. Statistical analysis revealed that burst strength value differences were significant between the Coll sample and materials with chitosan. On the other hand, the difference between Coll:Chit and Coll:Chit:HT was not significant. These results suggest that incorporating chitosan in the collagen material improved the sample’s mechanical resistance. The material with HT recorded a lower burst strength value than the Coll:Chit sample, which could suggest a plasticizing effect of this compound. However, this difference was not significant, indicating that HT’s presence showed no evident mechanical impact. These results agree with the literature, where several published works demonstrated the positive effect of chitosan in improving the mechanical robustness of collagen materials [46,47]. Nonetheless, a straightforward comparison is not easy because the tests implemented are not standardized; therefore, a direct comparison cannot be established. Regarding the distance at burst, a larger distance to achieve the material burst suggests a higher elastic material, whereas smaller distances suggest a more brittle behavior [48]. However, the texturometer analysis showed similar results between samples with no significant differences, suggesting that chitosan and HT did not contribute to a different elastic behavior of the materials. Overall, the results revealed a higher robustness of materials prepared with chitosan, with the same elastic behavior as the sample composed only of collagen.

Evaluating the mechanical properties of solid-state materials for topical applications provides important information, particularly for handling dry materials. Nonetheless, topical applications, particularly in wound management, may lead to exudate absorption, dramatically changing the material’s mechanical behavior from dry to hydrated. So, the viscosity, viscoelastic behavior, and adhesiveness of the hydrated materials were measured. The materials were characterized in two different mass:solvent proportions (1:1 *w*:*w* and 1:4 *w*:*w*) to mimic different fluid absorptions under two different wound fluid-draining conditions, from moderate (1:1 *w*:*w*) to copious (1:4 *w*:*w*) wound exudate volumes, after contact with the wounded skin area [49,50].

**Figure 3:**
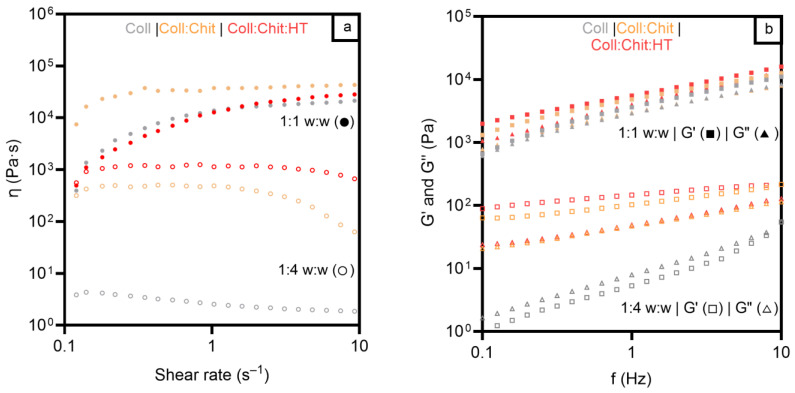
Viscosity measurements and shear rate (**a**) for all materials prepared with collagen (Coll), chitosan (Chit), and hydroxytyrosol (HT) in two different mass:solvent (*w*:*w*) hydrated states: 1:1 *w*:*w* (●) and 1:4 *w*:*w* (○). Frequency sweep test (**b**) for all materials prepared with Coll, Chit, and HT in two different mass:solvent (*w*:*w*) hydrated states: 1:1 *w*:*w* G′ (◼) and G″ (▲), and 1:4 *w*:*w* G′ (◻) and G″ (△).

Figure 3a presents the viscosity results of the three materials in the two hydrated states studied. As expected, all the samples revealed a higher viscosity in the 1:1 *w*:*w* condition due to the lower liquid content of the materials. Viscosity behavior, as a function of shear rate, showed similar profiles for all samples in a 1:1 *w*:*w* ratio, with viscosity increasing from 0.1 s^−1^ to 1 s^−1^ and remaining constant between 1 s^−1^ and 10 s^−1^. The Coll:Chit sample presented higher viscosities at a lower shear rate than Coll and Coll:Chit:HT samples, but tended toward similar values at a higher shear rate (20^4^–50^4^ Pa·s at 10 s^−1^). In contrast, the results of the tested samples under a 1:4 *w*:*w* ratio showed different viscosity profiles between samples with and without chitosan across all the tested shear rates. The samples containing chitosan exhibited viscosities at least two orders of magnitude higher than those composed solely of collagen. These results suggest that chitosan influenced the internal structure of the collagen materials, unlike HT, which did not change the materials’ viscosity in any of the tested conditions.

To further evaluate the mechanical properties of the prepared materials, the viscoelastic properties of the different hydrated materials were studied and are presented in Figure 3b.

Concerning the oscillation frequency test, all samples exhibited predominantly elastic behavior at a higher mass:solvent ratio, evident from the greater magnitude of the elastic modulus (G′) compared to that of the viscous modulus (G″). This indicates that the structure of the gels remained intact throughout the entire range of frequencies, confirming that all the hydrated materials present a strong network and a solid-like behavior (G′ > G″) [43]. For the characterization under a lower mass:solvent ratio, all samples presented lower values of the complex shear modulus. However, the samples containing chitosan (Coll: Chit and Coll:Chit:HT) presented a solid-like behavior. In contrast, a transition to liquid-like behavior is observed in the collagen sample since G″ became higher than G′ at higher hydrated treatment. These results agree with the purpose of chitosan incorporation in conferring higher robustness to collagen materials. Viscosity and viscoelastic assessment revealed that the presence of this polymer is required to maintain higher viscosity and preserve a gel structure even under increased water absorption conditions. These rheology test results also suggested an approximated shear-thickening behavior of all materials since the materials’ viscosity increased with the applied stress [51]. This behavior indicates that the collagen materials could maintain the structural integrity in a wound bed under some stress and provide consistent coverage and protection, as previously described in the literature [52].

Another essential aspect of the screening analysis of the material’s potential for wound healing applications concerns its adhesiveness. Materials must be self-supported on the wound; otherwise, there is a need for an additional support dressing. On the other hand, dressings must also be easy to remove, avoiding traumatic and painful dressing removal for the patient, which could compromise wound healing [4,5]. Therefore, a tack and pull-away test was carried out, and the results are shown in Table 4. This assessment enables the evaluation of a sample’s tackiness and adhesive strength by measuring the peak normal force and area under the force–time curve (with a larger area indicating a stronger adhesive) as two parallel plates are pulled apart [53]. Tackiness in the context of material behavior is associated with stickiness and may result from adhesive forces between two materials in contact [54,55].

As expected, the results revealed that samples with lower water content display more prominent stickiness and adhesive strength, with values of area under the force–time curve and absolute normal peak force up to 30 N·s and 15 N, respectively. However, the significant decrease in the sample’s stickiness with the 1:4 *w*:*w* treatment could be an interesting achievement. These results indicate that a reduction in material adhesiveness occurs at higher water absorption content, which could facilitate its removal painlessly for the patient. On the other hand, each treatment revealed similar results between samples, with slight differences that were not significant. These results suggest that incorporating this chitosan and HT content did not significantly influence the collagen material’s stickiness.

**Figure 4:**
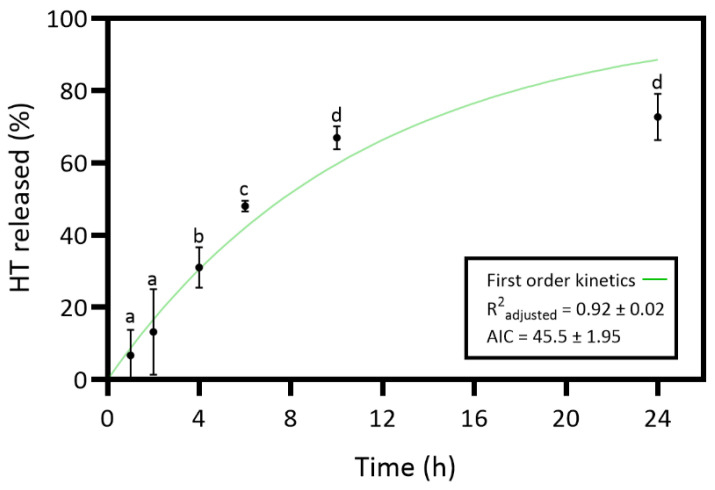
Release profile and the corresponding first-order model of hydroxytyrosol (HT) from the collagen (Coll)–chitosan (Chit)–HT biomaterial prepared. Statistically significant differences comparing all time points are indicated by different letters (a–d). (Mean ± SD, *n* = 4).

In this study, HT was incorporated into the collagen–chitosan matrix, expecting that the HT release into the wound environment would enhance the bioactive properties of the dressing material. In vitro release studies evaluated the HT release from the collagen–chitosan material throughout 24 h. The study aimed to understand the kinetic release profile of HT, and the results are presented in Figure 4.

The results of the HT release revealed an increasing release profile over time and a maximum release percentage (72.7%) at 24 h. However, the slight differences between the 24 h and 10 h time points are not statistically significant. So, the maximum HT release percentage would have already been at 10 h. The HT in vitro release was curve-fitted to zero-order (Equation (2)), first-order (Equation (3)), Higuchi (Equation (4)), and Korsmeyer–Peppas model (Equation (5)) to understand the release kinetics [18,56]. Controlled-release dosage forms are crucial for effective wound healing treatment, requiring an understanding of specific mass transport mechanisms for predicting quantitative kinetics and in vivo behavior. The polymer matrix content can be released through various mechanisms such as diffusion, erosion, and dissolution [56]. Table S1 presents the results of all tested models. The first-order model revealed an R^2^ _adjusted_ of 0.92 ± 0.02 and the lowest AIC value of 45.5 ± 1.95. Thus, the release kinetics results suggest that the HT dissolution profile from the hydrated collagen–chitosan matrix is concentration-dependent [56].

**Figure 5:**
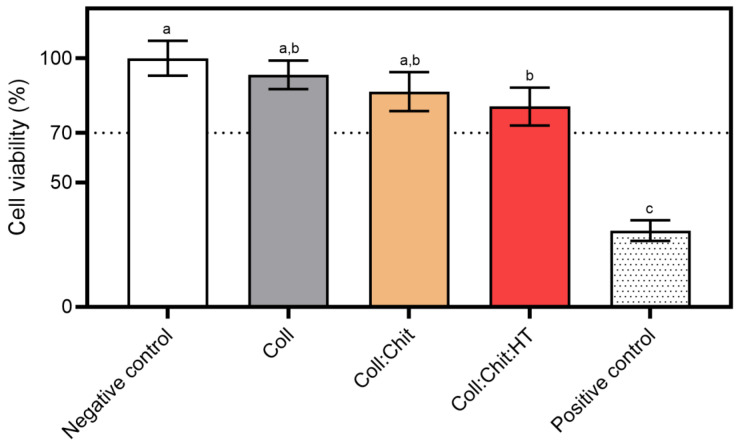
Cytotoxicity assay using the MTS reagent: materials prepared with collagen (Coll), chitosan (Chit), and hydroxytyrosol (HT) were incubated with the NCTC clone 929 cell line for 24 h at 37 °C and 5% CO_2_ humidified atmosphere (mean ± SD, *n* = 3). A 10% (*v*/*v*) solution of DMSO in cell culture media was used as a positive cytotoxic control. The samples demonstrate a cytotoxic potential if viability is reduced to < 70% of the negative control (untreated cells, where no sample or cytotoxic compound was added). Statistically significant differences comparing all conditions are indicated by different letters (a–c).

The present work aimed to develop an innovative material for potential biomedical applications involving direct contact with skin cells. The cytotoxicity of the produced samples on a mouse fibroblast cell line was assessed to screen the biocompatible safety of the produced materials for potential wound healing applications. Figure 5 presents the ISO 10993-5 direct contact methodology results, which involved placing the material in direct contact with cultured cells. This method simulates real-life interactions between the material and skin cells, allowing for the identification of potential toxic effects from the material or its leachable and predicting biocompatibility.

All tested materials present similar cell viability percentages. However, statistical analysis revealed significant differences in cell viability percentages between the negative control (non-treated cells) and the Coll:Chit:HT sample. This is the only sample containing HT, suggesting that the release of this compound could contribute to the significant decrease in cell viability. Nevertheless, none of the tested samples, including the Coll:Chit:HT sample, exhibited cell viability percentages below the potential cytotoxic effect threshold defined by the ISO 10993-5 standard (70%). In this work, the HT concentration incorporated into the material was based on the MIC value of this compound against both bacterial targets selected. However, future works could directly investigate HT cytotoxicity by measuring the half-effective and inhibitory concentrations (EC_50_ and IC_50_, respectively) if direct information regarding the cytotoxicity of HT is required.

After the materials were found to be non-cytotoxic, their antimicrobial activity was evaluated against *S. aureus* and *P. aeruginosa*. To determine the produced materials’ antimicrobial activity and assess the impact of incorporating chitosan and HT in the collagen matrix, the absorption method outlined in ISO 20743:2013 was performed. This method evaluates the microorganism’s reduction on the material’s surface by measuring the number of viable microorganisms after a specified contact period. It is particularly suitable for assessing the antimicrobial activity of materials intended for wound healing applications by simulating the material’s direct contact with the wound, which is potentially exposed to various pathogens, and due to its comprehensive and standardized approach. ISO 20743:2013 specifies *K. pneumoniae* as the surrogate Gram-negative bacterial target for the assay instead of *P. aeruginosa*. The scope of this standard is to assess textile antimicrobial efficacy, and the authors agree that *P. aeruginosa* is a more relevant and adequate representative of Gram-negative skin commensals and nosocomial pathogens. Table 5 shows the antimicrobial activity values according to the absorption method of ISO 20743:2013 and their corresponding efficacy.

Regarding *S. aureus*, the determination of antibacterial activity showed the efficacy of all tested materials. Even the sample composed solely of collagen demonstrated significant activity. On the other hand, Coll:Chit and Coll:Chit:HT presented strong antibacterial activity against *S. aureus*. Regarding *P. aeruginosa*, the Coll sample’s activity value was insufficient to be considered effective. In addition, although both samples containing chitosan revealed efficacy against *P. aeruginosa*. Only Coll:Chit:HT displayed strong efficacy. These results indicate that the antibacterial activity of collagen materials was improved with chitosan incorporation. Also, the higher efficacy against *P. aeruginosa* suggests that not only chitosan but also incorporating HT enhances the antibacterial activity of collagen materials. These results agree with the literature’s reported works and the MIC values in Table 2, where these compounds showed antibacterial activity against *S. aureus* and *P. aeruginosa* [36,37,38]. As previously discussed, some HT degradation can occur in the wound-healing fluid environment. However, the antimicrobial evaluation of aged HT solutions (up to 60 days; Table S2) suggests that materials incorporating this compound might preserve their activity against representative skin commensals and nosocomial pathogens, remaining effective over time.

## 4. Conclusions

Collagen–chitosan composites with HT were successfully prepared. The solid materials, obtained by freeze-drying a Coll:Chit:HT gel mixture, displayed a sponge-like structure with a macroporous network, a feature shared by all the prepared materials. Textural solid-state analysis revealed a positive influence of chitosan on the material’s mechanical resistance performance. Additionally, rheological studies suggested that chitosan incorporation preserved the material’s elastic behavior under a higher hydrated state. This study also indicated that a concentration-dependent mechanism released HT from the collagen–chitosan matrix with a controlled-release profile. Furthermore, bioassays proved the materials’ cytocompatibility with skin cells and strong antibacterial activity against *S. aureus* and *P. aeruginosa*. Incorporating chitosan and HT enhanced the collagen material’s antibacterial efficacy. In vivo studies are still necessary to assess the material’s wound-healing performance and validate the compound’s biocompatibility and bioactivity in biological models closer to their final application (e.g., pH and temperature variations for certain distinct phases). Future assessments of the material’s rheologic behavior and HT stability are needed to understand its behavior under varying wound conditions (e.g., pH and temperature). Additionally, advanced cell models or in vivo studies are necessary to validate the material’s wound-healing performance, biocompatibility, and bioactivity in relevant biological models. In conclusion, in this study, HT was successfully incorporated into collagen–chitosan composites, enhancing the promising therapeutic effect of the resultant material. Coll:Chit:HT presents a very promising potential as an advanced wound-healing material for various topical diseases and pathologies.

## Figures and Tables

**Table 1:** Compounds, dissolving agents, and respective highest-tested concentrations in the antimicrobial susceptibility testing assays.

Compounds	Dissolving Agent	Highest-Tested Concentration
Chitosan	HCl 0.1 M	10 mg/mL
Hydroxytyrosol	ddH_2_O	20 mg/mL
HCl	ddH_2_O	50 mM
Xylitol	ddH_2_O	300 mg/mL
Citric acid	ddH_2_O	100 mg/mL

ddH_2_O: Sterilized double-distilled water.

**Table 2:** Antimicrobial susceptibility testing assays against *Staphylococcus aureus* ATCC 6538 (WDCM 00193) and *Pseudomonas aeruginosa* ATCC 27853 (WDCM 00025) for chitosan (Chit) and hydroxytyrosol (HT).

Tested Compounds	MIC_Median_ (*n* = 1/*n* = 2/*n* = 3) (mg/mL)	
	*S. aureus* ATCC 6538	*P. aeruginosa* ATCC 27853
Chit	**0.16** (0.16/0.16/0.16)	**0.31** (0.63/0.63/0.31)
HT	**0.39** (0.39/0.39/0.39)	**1.56** (1.56/0.78/0.78)

The MIC values (mg/mL) are represented as the median (MIC_Median_) of three independent assays (*n* = 1/*n* = 2/*n* = 3).

**Table 3:** Textural analysis of the solid materials prepared with collagen (Coll), chitosan (Chit), and hydroxytyrosol (HT).

Samples	Burst Strength (N)	Distance at Burst (mm)
Coll	13.9 ± 1.82 ^a^	53.0 ± 0.40 ^a^
Coll:Chit	19.7 ± 3.57 ^b^	53.5 ± 0.20 ^a^
Coll:Chit:HT	17.1 ± 0.89 ^b^	53.5 ± 0.18 ^a^

Statistically significant differences comparing all samples in each parameter are indicated by different letters (a, b).

**Table 4:** Adhesive properties for all materials prepared with collagen (Coll), chitosan (Chit), and hydroxytyrosol (HT) in two different mass:solvent hydrated states: 1:1 *w*:*w* and 1:4 *w*:*w*.

	Area Under the Force–Time Curve (N·s)		Peak of Normal Force (N)	
	1:1 *w*:*w*	1:4 *w*:*w*	1:1 *w*:*w*	1:4 *w*:*w*
Coll	27.9 ± 2.26 ^a^	1.44 ± 0.54 ^b^	−15.4 ± 1.30 ^a^	−0.12 ± 0.07 ^b^
Coll:Chit	24.4 ± 1.76 ^a^	1.18 ± 0.54 ^b^	−12.4 ± 2.01 ^a^	−0.27 ± 0.13 ^b^
Coll:Chit:HT	25.1 ± 1.53 ^a^	1.24 ± 0.50 ^b^	−12.5 ± 1.87 ^a^	−0.22 ± 0.08 ^b^

Within each evaluated parameter (area under the force–time curve and peak of normal force), statistically significant differences comparing all samples and hydrated states are indicated by different letters (a, b).

**Table 5:** Antimicrobial activity values and corresponding efficacy of the tested materials against *Staphylococcus aureus* ATCC 6538 (WDCM 00193) and *Pseudomonas aeruginosa* ATCC 27853 (WDCM 00025) after contact with samples at a 2 g/mL concentration during 24 h at 37 °C.

Strain	Sample	Antibacterial Activity Value	Efficacy of Antibacterial Activity
*S. aureus* ATCC 6538	Coll	2.1 ± 0.3	Significant
	Coll:Chit	6.9 ± 0.1	Strong
	Coll:Chit:HT	7.1 ± 0.1	Strong
*P. aeruginosa* ATCC 27853	Coll	1.7 ± 0.2	Negligible
	Coll:Chit	2.6 ± 0.2	Significant
	Coll:Chit:HT	5.8 ± 0.5	Strong

Efficacy of antibacterial activity: strong (for antibacterial activity value ≥ 3); significant (for 2 ≤ antibacterial activity value < 3); negligible (for < 2 antibacterial activity value).

## Data Availability

The original contributions presented in this study are included in the article/Supplementary Material. Further inquiries can be directed to the corresponding author(s).

## Supplementary Materials

The following supporting information can be downloaded at: https://www.mdpi.com/article/10.3390/pharmaceutics17050618/s1, Table S1. Kinetic model results of hydroxytyrosol in vitro release studies; Table S2: Antimicrobial susceptibility testing assays against *Staphylococcus aureus* ATCC 6538 (WDCM 00193) and *Pseudomonas aeruginosa* ATCC 27853 (WDCM 00025) of the NADES constituents, compounds incorporated in collagen materials, and chitosan dissolving agent (HCl). Median and triplicate values of MICs are presented.
