# Supplementary material for: Collagen–Chitosan Composites Enhanced with Hydroxytyrosol for Prospective Wound Healing Uses
Source: Pharmaceutics. 2025 May 6;17(5):618. doi: 10.3390/pharmaceutics17050618 (PMC12114697; doi:10.3390/pharmaceutics17050618)
Supplement: Supplementary file 1 [file pharmaceutics-17-00618-s001.zip › pharmaceutics-3560602-supplementary.pdf]

Collagen–Chitosan Composites Enhanced with Hydroxytyrosol for Prospective Wound Healing Uses

Miguel P. Batista [1,2], Margarida Pimenta [1,3], Naiara Fernández [1], Ana Rita C. Duarte [2], Maria do Rosário Bronze [1,3,4], Joana Marto [5,*] and Frédéric Bustos Gaspar [1,3,*]

[1] iBET—Instituto de Biologia Experimental e Tecnológica, Apartado 12, 2781-901 Oeiras, Portugal; mbatista@ibet.pt (M.P.B.); margarida.pimenta@ibet.pt (M.P.); naiara.fernandez@ibet.pt (N.F.); mbronze@ibet.pt (M.d.R.B.)
[2] LAQV-REQUIMTE, Associated Laboratory for Green Chemistry—Network of Chemical and Technology, Departamento de Química, Faculdade de Ciências e Tecnologia, Universidade Nova de Lisboa, Quinta da Torre, 2829-516 Caparica, Portugal; ard08968@fct.unl.pt
[3] Instituto de Tecnologia Química e Biológica António Xavier, Universidade Nova de Lisboa, Av. da República, 2780-157 Oeiras, Portugal
[4] FFULisboa, Faculty of Pharmacy, Universidade de Lisboa, Av. das Forças Armadas, 1649-019 Lisboa, Portugal
[5] Research Institute for Medicines (iMed.ULisboa), Faculty of Pharmacy, Universidade de Lisboa, Av. das Forças Armadas, 1649-003 Lisboa, Portugal
* Correspondence: jmmarto@ff.ulisboa.pt (J.M.); fgaspar@ibet.pt (F.B.G.)

Supplementary material

Table S1. Kinetic model results of hydroxytyrosol *in vitro* release studies.

Model	k	$R^2_{adjusted}$	AIC
Zero-order	3.90 ± 0.27	0.57 ± 0.02	56.0 ± 0.29
First-order	0.09 ± 0.01	0.92 ± 0.02	45.5 ± 1.95
Higuchi	16.5 ± 1.12	0.87 ± 0.04	47.3 ± 2.64
Korsmeyer-Peppas	16.2 ± 2.07	0.85 ± 0.05	49.2 ± 2.61

Table S2. Antimicrobial susceptibility testing assays against *Staphylococcus aureus* ATCC 6538 (WDCM 00193) and *Pseudomonas aeruginosa* ATCC 27853 (WDCM 00025) of the NADES constituents, compounds incorporated in collagen materials, and chitosan dissolving agent (HCl). Median and triplicate values of MICs are presented.

Tested compounds		MIC_{Median} (n=1/n=2/n=3)	
		S. aureus ATCC 6538	*P. aeruginosa* ATCC 27853
Citric acid (mg/mL)		1.56 (1.56/1.56/1.56)	3.13 (3.13/3.13/3.13)
Xylitol (mg/mL)		300 (300/300/300)	300 (300/300/300)
HCl (mM)		12.5 (12.5/12.5/12.5)	25.0 (25.0/25.0/25.0)
Chitosan (mg/mL)		0.16 (0.16/0.16/0.16)	0.63 (0.63/0.63/0.31)
Hydroxytyrosol (mg/mL)	Fresh solution	0.39 (0.39/0.39/0.39)	0.78 (1.56/0.78/0.78)
	Aged solution, 1 day at 37 ºC	0.39 (0.39/0.39/0.39)	0.78 (0.78/0.78/0.78)
	Aged solution, 60 days at 4ºC	0.20 (0.20/0.20/0.20)	0.78 (0.78/0.78/0.78)
